# Qual o Papel dos Biomarcadores de Lesão Renal na Nefropatia Induzida por Contraste?

**DOI:** 10.36660/abc.20210433

**Published:** 2021-06-08

**Authors:** Pedro Pimenta de Mello Spineti

**Affiliations:** 1 Hospital Universitário Pedro Ernesto Rio de JaneiroRJ Brasil Hospital Universitário Pedro Ernesto , Rio de Janeiro , RJ - Brasil; 2 Hospital Unimed-Rio Rio de JaneiroRJ Brasil Hospital Unimed-Rio , Rio de Janeiro , RJ – Brasil

**Keywords:** Lesão Renal Aguda, Nefropatias, Meios de Contraste, Biomarcadores, Cateterismo Cardíaco, Creatinina, Lipocalina 2

A lesão renal aguda induzida por contraste (LRA-IC) é uma complicação potencial e importante na utilização de meios de contraste radiológicos iodados estando associada com maiores taxas de morbimortalidade e aumento no tempo de hospitalização em pacientes submetidos a cateterismo cardíaco. ^1^ Sua incidência é variável na literatura de acordo com o critério utilizado para o seu diagnóstico. A definição LRA-IC mais frequentemente utilizada em ensaios clínicos é uma elevação nos níveis de creatinina sérica (Cr) de 0,5 mg/dl ou de 25% em relação ao valor basal, em até 72 h após a exposição ao meio de contraste. ^1^

No entanto, a Cr apresenta uma série de limitações como marcador da função renal. Seu nível sérico é influenciado por fatores externos como sexo, idade, cor da pele, peso e massa muscular. Ela subestima a função renal em mulheres, pessoas idosas ou com baixo peso. Sua variação superestima o dano renal em indivíduos com disfunção renal prévia. Outra importante limitação deve-se ao fato de a Cr elevar-se somente após 24 h de uma lesão renal aguda, sendo considerada um “marcador lento” de injúria renal aguda. ^1 , 2^

Novos biomarcadores têm sido avaliados no auxílio ao diagnóstico da LRA-IC. Entre estes, destacam-se a cistatina C (Cis-C), a lipocalina associada à gelatinase de neutrófilos (NGAL) e a molécula de injúria renal 1 (KIM-1).

A Cis-C é um peptídeo de 122 aminoácidos com baixo peso molecular (13,36 Kda), da família dos inibidores de protease da cisteína. Ela é produzida de forma constante pela maioria das células nucleadas e sua síntese não é influenciada por processos inflamatórios, pela massa muscular ou pelo sexo do indivíduo. Devido ao seu baixo peso molecular e à carga positiva, ela é livremente filtrada pelo glomérulo renal e, então, reabsorvida e metabolizada no túbulo renal proximal, não ocorrendo secreção renal ou extra-renal. Logo, sua determinação sérica reflete, exclusivamente, a filtração glomerular e seu aumento no soro significa uma redução dessa taxa. ^2^ A Cis-C atinge seu pico 24 h após a exposição ao contraste em pacientes com LRA-IC e permanece elevada até 48 hs. ^3^

A NGAL é uma glicoproteína de 178 aminoácidos que pertence à superfamília das lipocalinas. Ela é expressada pelos neutrófilos e por certos epitélios, como os túbulos renais. Ela é filtrada livremente pelo glomérulo e posteriormente reabsorvida pelas células do túbulo proximal. Seus níveis séricos e urinários basais são muito baixos, elevando-se em diversos cenários clínicos como, inflamação sistêmica, câncer e aterosclerose. ^2^ Seus níveis elevam-se agudamente 4 h após exposição ao contraste em casos de LRA-IC e retornam aos níveis basais em 48. ^3^

Revisão sistemática recente sobre o papel da NGAL e da Cis-C analisou 37 estudos e concluiu que ambas podem servir como indicadores diagnósticos precoces de LRA-IC, e que a cistatina C pode ter um desempenho melhor do que a NGAL. Não houve diferença no desempenho da NGAL sérica, comparada à urinária. ^4^

A KIM-1 humana é uma glicoproteína transmembrana do tipo um, com um domínio de imunoglobulina e mucina que não é detectável em tecido renal normal ou na urina, mas é expresso em níveis muito elevados em células desdiferenciadas do epitélio tubular proximal renal após lesão isquêmica ou tóxica. Há inúmeras características que poderiam torná-la um atraente biomarcador de lesão renal, tais como: ausência em rim normal, aumento da sua expressão após um insulto isquêmico agudo e sua persistência nas células do epitélio tubular até sua recuperação completa. ^2^

Nesta edição dos Arquivos Brasileiros de Cardiologia, o Dr. Huyut ^5^ avaliou a associação entre os níveis séricos de KIM-1 e LRA-IC em pacientes idosos com infarto do miocárdio sem supradesnivelamento do segmento ST. Apesar do pequeno tamanho da população estudada, ele pode demonstrar que esta molécula esteve associada de forma independente à LRA-IC com uma boa área sob a curva-ROC. A LRA-IC, como esperado, esteve associada a maior morbimortalidade.

Embora o achado seja interessante, dois estudos recentes comparando NGAL e KIM-1 demonstraram que esta parece ter uma performance pior na predição de LRA-IC. ^6 , 7^ Estas divergências de resultados podem ser atribuídas à diferentes definições de LRA-IC utilizadas pelos estudos, assim como pontos de corte distintos para os biomarcadores.

Em conclusão, os novos biomarcadores apresentam vantagens em relação à creatinina para avaliação de LRA-IC, porém ainda há incerteza quanto ao melhor deles para esta indicação. São necessários novos estudos que avaliem não só a associação entre biomarcadores de LRA-IC, mas também a custo-efetividade da incorporação à prática clínica diária.
