# Supplementary material for: Qual o Papel dos Biomarcadores de Lesão Renal na Nefropatia Induzida por Contraste?
Source: Arq Bras Cardiol. 2021 Jun 8;116(6):1057–8. [Article in Portuguese] doi: 10.36660/abc.20210433 (PMC8288534; doi:10.36660/abc.20210433)
Supplement: Translation

# Translation: What Is the Role of Kidney Injury Biomarkers in Contrast-Induced Nephropaty?

**Authors:** Pedro Pimenta de Mello Spineti

**Affiliations:** 1 Brazil Hospital Universitário Pedro Ernesto, Rio de Janeiro, RJ - Brazil; 2 Brazil Hospital Unimed-Rio, Rio de Janeiro, RJ - Brazil

**Keywords:** Acute Kidney Injury, Kidney Diseases, Contrast Media, Biomarkers, Cardiac Catheterization, Creatinine, Lipocalin 2

Contrast-induced acute kidney injury (CI-AKI) is a potential severe complication in the use of iodinated radiological contrast media and is associated with higher rates of morbidity and mortality and increased length of hospital stay in patients undergoing cardiac catheterization.^1^ Its incidence is variable in the literature according to the criteria used for its diagnosis. The CI-AKI definition most often used in clinical trials is an increase in serum creatinine (Cr) levels of 0.5 mg/dl or 25% over baseline within 72 h after exposure to contrast medium.^1^

However, Cr has a number of limitations as a marker of renal function. Its serum level is influenced by external factors such as sex, age, skin color, weight and muscle mass. It underestimates renal function in women, in the elderly or in underweight individuals. Its variation overestimates renal damage in individuals with previous kidney failure. Another important limitation is the fact that Cr rises only after 24 h of an acute kidney injury, being considered a “slow marker” of acute kidney injury.^1 , 2^

New biomarkers have been evaluated to help diagnose CI-AKI. These include cystatin C (CysC), the lipocalin associated with neutrophil gelatinase (NGAL) and kidney injury molecule 1 (KIM-1).

CysC is a peptide of 122 amino acids with low molecular weight (13.36 Kda), from the family of cysteine protease inhibitors. It is produced steadily by most nucleated cells and its synthesis is not influenced by inflammatory processes, muscle mass or sex of the individual. Due to its low molecular weight and positive charge, it is freely filtered by the renal glomerulus and then reabsorbed and metabolized in the proximal renal tubule, with no renal or extra-renal secretion. Therefore, its serum determination reflects exclusively glomerular filtration and its increase in serum means a reduction in this rate.^2^ CysC reaches its peak 24 h after exposure to contrast in patients with CI-AKI and remains high for up to 48 h.^3^

NGAL is a 178 amino acid glycoprotein that belongs to the superfamily of lipocalins. It is expressed by neutrophils and certain epithelia, such as renal tubules. It is freely filtered by the glomerulus and later reabsorbed by the cells of the proximal tubule. Its basal serum and urinary levels are very low, rising in different clinical settings such as systemic inflammation, cancer and atherosclerosis.^2^ Its levels rise sharply 4 h after exposure to contrast in cases of CI-AKI and return to baseline levels in 48h.^3^

A recent systematic review of the role of NGAL and CysC analyzed 37 studies and concluded that both can serve as early diagnostic indicators of CI-AKI, and that cystatin C may perform better than NGAL. There was no difference in the performance of serum NGAL compared to urine NGAL.^4^

Human KIM-1 is a type one transmembrane glycoprotein, with an immunoglobulin and mucin domain that is not detectable in normal renal tissue or in the urine, but it is expressed at very high levels in dedifferentiated cells of the renal proximal tubular epithelium after ischemic or toxic injury. There are numerous characteristics that could make it an attractive biomarker of kidney injury, such as: absence in normal kidney, increased expression after an acute ischemic insult and its persistence in the tubular epithelium cells until its complete recovery.^2^

In this edition of Arquivos Brasileiros de Cardiologia, Dr. Huyut^5^ evaluated the association between serum levels of KIM-1 and CI-AKI in elderly patients with ST-segment elevation myocardial infarction. Despite the small size of the study population, he shows that this molecule was independently associated with CI-AKI with a good area under the ROC curve. CI-AKI, as expected, has been associated with increased morbidity and mortality.

Although this is an interesting finding, two recent studies comparing NGAL and KIM-1 demonstrated that it appears to have a worse performance in predicting CI-AKI.^6 , 7^ These inconsistent results can be attributed to the different definitions of CI-AKI used by the studies, as well as different cutoff points for the biomarkers.

In conclusion, the new biomarkers have advantages on creatinine for the evaluation of CI-AKI, but there is still uncertainty about the best of them for this indication. Further studies are needed to assess not only the association between CI-AKI biomarkers, but also the cost-effectiveness of incorporating them into daily clinical practice.
